# Chicago Medical Society

**Published:** 1884-02

**Authors:** 


### CHICAGO MEDICAL SOCIETY.

The Chicago Medical Society held a well attended meeting on the evening of November 5, and listened to the reading of an able paper by Dr. A. Reeves Jackson on the question, "Is Extirpation of the Cancerous Uterus a Justifiable Operation ?" The paper is essentially the same as presented by the author at a recent meeting of the American Gynæcological Society, and synopsized briefly is as follows :

"Diagnosis of uterine cancer cannot be made sufficiently early to ensure its complete removal by extirpation of the uterus. When the diagnosis can be established there is no reasonable hope for a radical cure, and other methods of treatment, far less dangerous than excision of the entire organ, are equally as effectual in ameliorating suffering, retarding the progress of the disease, and prolonging life. Extirpation of the cancerous uterus is a highly dangerous operation, and neither lessens suffering, except in those whom it kills, nor gives reasonable promise of permanent cure in those who recover. Hence it fails in all the essentials of a beneficial operative procedure, and should not be adopted in modern surgery. The growing tendency to bold, fearless—may I not say reckless—progressiveness in the surgical branches of our profession, would have appalled our predecessors. When we consider that some of these achievements are scarcely more than antemortem examinations, whose chief usefulness consists in demonstrating how long their owners are able to survive the loss of certain bodily organs, we ask whether there is to be any limit to these exhibitions of surgical temerity? The removal of the whole uterus is not a very novel operation. Andreas A. Cruce removed the organ per vaginam for scirrhus in 1560. Similar operations were done in the eighteenth century by Wrisburg and by Monteggia. Blundell operated in three cases in 1828, two of the patients dying, and one surviving a year, and finally dying from a recurrence of the disease.

"In 1878 Prof. W. A. Freund reported a new method, under proper antiseptic precautions, whereby the uterus could be, as he thought, more safely removed than hitherto. In the early part of 1879 he had operated in ten cases, with the result of five deaths and five recoveries; and in September of that year, at the International Medical Congress at Amsterdam, he reported four additional cases of his own. In one of these, the operation was unfinished; the other three were fatal. A later table of operations by Freund's method has appeared, including 91 cases, of which 66 died; 25 recovered; mortality, 72.5 per cent. Yet at the London Congress, Freund made the astounding statement that the operation may be undertaken as a not very dangerous one in the early stages of sarcoma and carcinoma, in which it gives promise of a radical cure. In consequence of the frightful mortality following the abdominal method, Czerny, Schroeder, Martin and others have practiced the removal of the uterus by the vagina, and thus far with better results. A table compiled by Sänger (*Archiv für Gynäkologie*, Berlin, 1883) includes 143 patients, of whom 72 per cent. recovered and 28 per cent. died. Extirpation of the uterus for cancer does not save, but destroys life. In order to show how much life has been sacrificed by it, I accept all the known fatal operations as the full number, although it is certain that there have been many more. They amount to 157 cases, 97 by the abdominal and 60 by the vaginal method.

If we grant that in all these cases the disease affected the cervix, and that the average length would be seventeen months, the calculation would show more than 222 years of life—over two centuries—sacrificed by the operation. If we consider that in many of the cases the disease affected the corpus uteri, as it surely did, in which the average duration of life is two and a half years, the aggregate amount of life destroyed would be even greater. But the writer is assured that 70 per cent. of lives are destroyed within a few hours, and 50 to 75 per cent. of the number that survive the operation, die from recurrence of the disease within a few months, and the few others who apparently receive benefit from an operation die as soon as though no operation had been performed."

In the discussion, in which quite a number participated, Dr. E. Andrews asked if the tumors of the cervix alluded to in the paper, were the scirrhus or epithelial form? Bilroth reports 11 per cent. of removal of tumors from the breast, and 33 per cent. of the lips and rectum, are successful if they are epithelial in nature.

Dr. W. E. Clarke said in his experience all patients operated upon in the cervix died within a year. One case of removal of a breast, he said, was done in 1861, and the lady recovered and remained so for nineteen years, but two years ago died of the disease. In his observation of 16 cases of amputation of the breast, all died.

Dr. R. H. Engert reported a case of cancer of the anterior lip of the cervix operated upon three years ago. The patient recovered, and at present there is no recurrence of the disease.

Dr. E. C. Dudley agreed with the essayist but thought cancer might and ought to be removed when it appears in other parts of the body, and recited a case he operated on four years ago of a cancerous tumor of the pelvis. The patient is a friend of his family, and there is no sign of a recurrence of the disease. The tumor was at once (directly after removal) examined with a microscope, and cancer cells were discovered in abundance. Some tumors occupy the middle ground bordering on the malignant, and yet they are benign. Another case he cited was where the tumor had ruptured five times in the peritoneal cavity, bringing on peritonitis each time. He operated, and applied thirty to forty ligatures. He used antiseptic precautions, and she recovered. This tumor was a cyst, containing a great deal of solid tissue. It was an ovarian tumor, and yet it was what might be called an endogenous cancer. The patient was operated on three years ago, and she is in perfect health to-day; and it appears to be a permanent cure, at least it is so up to this date. But if the neighboring glands are involved, as the breast and axilla and under the clavicle, then it is a serious question about operating with a hope of cure. The uterus offers a very unpromising field for operating or extirpation, as all patients die. As the fallopian tubes are a part of the uterus, and these are not removed in operations for sarcoma of the uterus, we should not decide positively no to.

Dr. G. C. Paoli had seen many operations of this kind; two were in Ohio, and one in Chicago; some died before the operation was completed; some died in a few days, and one lived six weeks. In cancer of the breast, sometimes the tumors are proved to be fibrous; he had seen two cases of this variety in the hospital in his native country, and they both recovered, and the surgeon acknowledged them to be fibrous in character, but in true cancer it is sure to return, either of the breast or the uterus.

Dr. R. H. Engert said if a tumor of the breast is decidedly pronounced to be a cancer, she would have it operated upon and removed.

Dr. A. H. Tagert spoke of a case operated on for removal of cancer of the breast twelve years ago, and the patient is living in this city, and with no appearance of the disease returning.

Dr. D. T. Nelson hardly coincided with the sweeping statement of the paper. He thinks there is a border line between the severely malignant, or epithelioma, and sarcoma. He would operate upon sarcoma, and thought possibly it would not return, but in carcinoma, he thought there was little hope of its not returning, when removed by the knife. He thinks this disease begins as a local disease at first, and if we could diagnosticate the case early, we might operate, and if it is sarcoma, it might sometimes be cured.

Spencer Wells, up to 1881, had never operated upon a carcinomatous uterus.

Dr. Williams thought we should remove the cervix, and so much of the cervix and body of the womb as we can see to be involved, then treat the wound with bromine. He reported that some cases recovered. The vaginal method is the safest, and offers greater permanency of cure, but the broad ligaments are left in operating, so, if we can diagnose the case early, we are justified in removing it. One case of a cancer of the breast the removed ten years ago, and the lady is now living. Another case was that of a soldier, whose axillary glands were deeply involved, also the artery. The surgeons desisted from cutting deeper, for fear of death. Hospital gangrene set in, the parts sloughed off, and the man was for two years afterward in the invalid corps performing duty, and he thought, in operations for either epthelioma or sarcoma, that the chance for recovery was more favorable than that of carcinoma.

Dr. Jackson, in closing the discussion, said he feared, from some of the remarks that had been made, that he had failed to make himself understood. He believed in the local origin of cancer, and believed in its removal, if removal be possible. He objected, however, to operations which destroy more than 50 per cent. of lives, and which experience has shown do not remove the disease in the cases of those who recover. In operations for cancer, the object is not to remove a mammary gland, a pylorus, or a uterus—it is to cure a *disease ;* and if this be not done, the operation is a failure. It has not done what it aimed to do, and it is none the less a failure because the patient may survive without the ablated organ for a few weeks or a few months. He had only discussed the question as to the feasibility of extirpation of the entire uterus for cancerous disease, an operation shown to be much more dangerous than the disease itself. He approved of the minor and safer methods—the curette, cautery, caustics, vaginal or supra-vaginal amputation, etc., because they were capable of doing all that could be usefully done by total excision, with comparatively little danger to life. In conclusion, he would mention a fact that was rather humiliating to surgeons, namely, that the greatest success in removal of the uterus had been obtained by midwives. There were on record no less than six cases in which that organ had been forcibly dragged from the pelvis, with but a single death.

At the same meeting, Dr. J. Elliott Colburn read an interesting paper on the "Treatment of Trichiasis by Electrolysis," which was of much interest to ophthalmologists. He has treated 54 cases by this method at the Eye and Ear Infirmary and Central Free Dispensary, during the past six months, with decided improvement in about every case. The subject, while not of great interest to the general practitioner, is none the less valuable, as the many points of interest it contained are a fund of information to those making diseases of the eye a specialty; even by those specialists the operation has not been extensively practiced.

There was considerable discussion on the subject of the formation of a "Nucleus for a Medical Library." A surplus of $500 was in the treasury of the society, which might be appropriated at once for this purpose. Further discussion was postponed, but no doubt this scheme will meet with an affirmative vote ere long.

The society adjourned at a late hour. L. H. M.

---

The Chicago Medical Society held a regular semi-monthly meeting on the evening of December 17, 1883.

Dr. D. W. Graham presided. The scientific business transacted consisted in a disposal of two papers. The first being a paper on "Alveolar Abscess opening into the Antrum and Nasal Cavity, Simulating Chronic Nasal Catarrh," was read by Dr. John S. Marshall. It contained the following points of interest, regarding the origin and causes of ozæna, namely syphilis, struma, lupus, ulceration, caries of the bones or cartilages, inflammation of the mucous membrane of the frontal and maxillary sinuses, and alveolar abscess opening into the antrum of Highmore or the nasal cavity direct.

The origin of certain forms of chronic nasal catarrh with fetid muco-purulent discharges is not always easy to locate, and especially is this true if the latter enumerated cause is the primary seat of trouble, and opens into the maxillary sinus. Abscesses forming at the apex of the roots of the superior incisor teeth, bicuspids, or molars, may discharge into the cavities of the antrum, or nasal fossa, and range from a slight nasal discharge to a most offensive ozæna, and it is not at all uncommon to find the roots of the superior molars penetrating the maxillary sinus. Less frequently, however, the bony plate over the apex of the incisors may likewise give way, especially over the apex of the central incisor, this may occur and an offensive discharge into the nasal passages or posterior nares may so closely simulate chronic nasal catarrh as to entirely mislead the most careful diagnostician, whereas the real source of trouble may be a diseased tooth. During the past three years the writer has seen two patients, who were suffering from this form of disease, who had been treated for months as cases of chronic nasal catarrh. The sphenoidal and frontal sinuses and the maxillary antrum may each be the seat of this morbid condition and fetid discharges from the nasal passages be the result, i. e. of the accumulation of the morbid products in the accessory cavity, which becomes distended, and the overflow escapes from the little orifice into the nasal cavity. Text-books allude to this disease in a mere paragraph sometimes, and undoubtedly the subject deserves more attention than has hitherto been given to it.

Antral abscess is a much more serious difficulty than the affection just described, the former causing, at its inception, rigors and fever, and involves much more of the adjacent structures, periosteum, etc. In antral abscess the pus may be retained between the periosteum and the bone, causing absorption of the walls of the antrum, and may open into the mouth, orbit, or through the external surface, and cause hideous deformity, or extensive necrosis of the maxilla often follows, and it may extend to the ethmoid, lachrymal, palatine, and inferior turbinated bones, with cerebral abscess, unless early recognized and interfered with by surgical means.

When a dental abscess opens into the maxillary sinus, there may be danger of septic poisoning if the secretions become foul and no other outlet is present. If this should occur it will be attended, of course, by serious constitutional symptoms. When it escapes into the nose the discharge will be unilateral, in its always being from the same nostril, and most abundant when a patient is lying upon the opposite side.

After the cases, with the symptoms, history, etc., had been cited, the paper concluded with the treatment, which consists in syringing the cavity of a tooth and the abscess through the pulp canal with warm carbolized water, 2 grs. to the ounce, once daily, and packing the tooth with carbolized cotton, and sealing it in with gutta percha stopping to exclude external moisture. Sometimes the buccal roots can be explored with a fine probe, by passing it upwards for an inch or inch and three-quarters. Water can be thrown into the antrum of Highmore through the roots of the teeth, and the fluid will escape into the nose. A tooth always needs to be extracted, if it is not already absent. Then we will discover, most likely, the pus escaping into the mouth through the alveoli, and that the ends of the buccal roots are somewhat rough, and portions already have been removed by absorption. A few weeks' time of treatment usually results most satisfactory.

The paper elicited considerable discussion. Dr. T. W. Brophy said that he had seen and treated a great many cases similar to those cited by the writer of the paper, that had previously been treated by physicians for post nasal catarrh ; he also illustrated his remarks by giving a method by which he was treating a case at present, and draining the antrum through an opening into the mouth by a metal tube or canula. The patient has been under his observation for ten months, and was for four months previously treated for the nasal difficulty alone by a physician having a large practice. The case is now almost entirely well. I think the discharge of pus into the nasal cavity occurs more frequently from the incisor teeth than from the molars, and necrosis frequently results from the alveolar abscess that at first has formed at the roots of the teeth, and sometimes involves the entire upper maxilla. He was much pleased with the paper, and had been much interested thereby.

Dr. Edmund Andrews inquired of the author what form of syringe he used in treating this class of cases, and what form of point the syringe had.

Dr. W. W. Allport stated that the paper presented by Dr. Marshall was well prepared and timely. That similar cases to those mentioned by him do sometimes occur, there is no doubt, but not so frequently that any one individual sees and treats a great many of them, as Dr. Brophy states he has done. I think that I am an ordinarily careful observer, but during my thirty-five years of practice I have seen but very few of such cases, not to exceed five or six, and I can hardly see how it is that any one man has treated so many cases similar to those mentioned in the paper, as the gentleman preceding me would have us infer that he has. Dentists frequently have fistulous openings and occasionally a diseased antrum to treat, caused by alveolar abscess. But alveolar abscess simulating nasal catarrh is met with but seldom, and I think Dr. Brophy has misunderstood the idea conveyed in the paper.

Dr. Robert Tilley stated that he had treated catarrhal troubles of the ear that had been primarily connected with the teeth, but a case had never occurred in his practice of catarrh of the nose originating from the teeth where perforation had occurred, as was described in the paper, and he took pains to examine the teeth and mouth carefully in making his diagnosis. He would like to inquire, without naming any one, if dentists had ever tried using the remedy peroxide of Hydrogen in "syringing out" this kind of pus cavity? He knew it to be an efficacious remedy for dentists to use, and suggested it as a valuable agent in the treatment of alveolar abscess. It consists of twelve volumes of oxygen in solution, and is used in full strength by oculists in Paris in treating eye affections, and he presumed it could be used in the same strength in treating these abscesses.

Dr. Brophy arose to add to his former remarks that he did not wish to be understood as saying boastfully that he had seen more cases of alveolar abscess than other dentists, as might be inferred from the remarks of one of the gentlemen who had the floor a few moments ago. He, however, would verify the statement he made at the beginning of the discussion: That he had met with a goodly number of cases of this variety, and he would at an early period present a paper on this subject before this society, which he would be pleased to do as soon as he can have the drawings made to illustrate the facts.

Dr. Allport said, regarding the use of peroxide of hydrogen, it is coming into general use by dentists. He knows it to be an exceedingly useful remedy as he has practiced using it a good deal, and concluded by saying: The author of the paper he thought, had ably called attention to an important subject this evening, one that is generally overlooked in diagnosing nasal catarrh.

Dr. Marshall closed the discussion by describing the kind of syringe used, as one speaker had inquired about, by saying it was the ordinary dental syringe fitted with a flexible metal point. stated that the peroxide of hydrogen is in general use, although he had not used the remedy in the treatment of this class of cases; but certainly should regard it to be quite valuable in the treatment of blind alveolar abscesses. Regarding the cases he had reported he said they had previously been under the observation of intelligent and well educated physicians. The object of the paper was to call the attention especially of the medical profession to one of the most obscure causes of fœtid nasal catarrh—a cause which in a majority of cases he thought was overlooked by physicians.

The following is the substance of a paper presented by Dr. Wm. L. Axford, on "Packing-House Wounds," from observations he made during the past three years. This interesting and important topic was attentively heard, for it involves our attention as surgeons in considering the best methods to secure primary union in wounds which admit of the possibility of such union, and the execrable manner in which this class of incised wounds heal, though cleanly cut by the knives that are used in packing-houses, and the bad consequences ensuing from very slight wounds on the hands and forearm, oftentimes amounting to a mere scratch, are familiar to many of you, and the sequelæ following are in proportion to the depth of the wound, for when the skin has been cut through and the cellular tissue opened, an intense cellulitis terminating in the formation of large quantities of pus, requiring free incisions has been almost invariably the result. From a small cut in the back of the hand, a cellulitis extending half way up the forearm is not an uncommon result, and cases under observation have occurred where the inflammation extended to the elbow. If the sheath of a tendon be opened, or the tendon cut, sloughing is the usual termination. If the wound extends deeper, and the periosteum be cut, nearly every case will be followed by periostitis with necrosis. The cause must, of course, be the septic material conveyed into the wound from knives used daily in cutting meat, which, probably, are rarely cleaned with care, and never disinfected, although they may be ground and washed daily. Wounds are of daily occurrence at some of these large houses, and from past experience I am led to think that we may expect the worst consequences to follow from the knives used by "shavers," *i. e.*, those employed in removing the finer hair from the bodies of the animals after they have passed through the "scraping machines,"(the "American hog," of course, being the animal alluded to that undergoes this sudden and complete metamorphosis.)

The wounds seem to pursue the same destructive tendency and resist antiseptic measures, as if no treatment were adopted. The treatment of wounds of this nature is, when necessary, to approximate the edges with sutures, then using freely either carbolic acid, solutions of encalyptol, or iodoform, all of which have been tried. Still union by the first intention has never yet occurred in the practice of the writer. A better plan might be to put aside the idea of securing union by the first intention, which is but problematical, and direct our measures towards preventing the inflamatory consequence by arresting the spread of the septics through the tissues, or, in other words, treat these cuts from the first as poisonous wounds, and try to prevent the further invasion of the septic material. To do so, if called immediately, apply thoroughly to the cut the actual cautery as the best means of destroying the poison. This heroic method, the writer is convinced, will diminish the suffering of a patient, and lessen the actual inconvenience so far as time of recovery goes. Perhaps, if we inject antiseptics, such as carbolic acid, tincture of iodine, or other solutions into the tissues about the wound they might take the place of the actual cautery, but their efficacy as adjuvants to the treatment by the cautery in fresh wounds can be better tested by using them after the more heroic method of searing the surface first has been done.

If, however, some time has elapsed since the injury before we are called, it would be more advisable to dispense with the cautery and trust entirely to the injections, and environ the wound by a layer of some reliable antiseptic dressing.

Among those who discussed the paper was Dr. E. Andrews, who stated that he was frequentlv called to attend severe cases, the result of so-called "packing-house wounds." Some patients are more violently poisoned than others; he recalled a case of a man whose foot had been injured by a machine that is used in an "offal factory" to grind the bones of the animals slaughtered there, and the man's foot was completely destroyed. The leg was at once amputated, but in twelve hours the flaps mortified, and the lower half of the thigh was distended with gas; in another twelve hours the entire thigh was tympanitic, and in a few hours more the man died, not so much from shock as from being poisoned to death. Another case he saw was where a man had been cut by a cleaver (used in packing-houses) on the lower portion of the "shin"; it pursued the same course to a fatal termination. The juices that oozed from the stump after he amputated the leg contained large numbers of bacteria, and the case presented what he termed septicæmia with a vengeance. He also gave the history of a third case with the same final disastrous result—death. Some authors termed this traumatic gangrene, others, gangrena septica. It is a virus, but I do not think many of the hogs have this virus.

Dr. Robert Tilley thought that no advantage could be gained by using a cautery in these cases if amputation proved to be of no avail, and he thought a simple hot poultice would be the better method to destroy the septicism, because bacteria do not thrive at a temperature a little above that of the body, or to keep a hot appliance continually to the wound. He had treated a number of cases of wounds received in soap factories, none of which had septicæmia.

Dr. Graham had seen a number of chronic cases of the kind of which the paper treated, and thought part of the virulence is due to the way this class of people live, as they live in a vitiated atmosphere at their homes, also the peculiar modes of the individuals in their hygienic care of themselves.

Dr. Axford thinks the cause of these poisonings is due to the knife being dipped in blood nearly constantly by butchers, and it instead of the hog, is the carrier of the virulence, at least in most of the cases; he is assured of this, and we should be on the alert for the after consequences which are most sure to arise, as he is convinced that every man wounded at these places by the knives used there, is actually poisoned.

L. H. M.

---

DR. HORATIO C. WOOD has at length severed his connection with the *Philadelphia Medical Times*, and its management now falls into the hands of Dr. Frank P. Woodbury, of Philadelphia. Dr. Woodbury is well known to the profession of the country at large as a gentleman who by education and experience as a journalist, is well qualified to sustain the duties which his distinguished predecessor has laid upon his shoulders. The *Philadelphia Medical Times* is one of the most valuable of American medical journals, not merely in the excellence of the editorial work it betrays, but in the character of those who contribute to its columns.

THE PREVENTION OF HORSE ACCIDENTS.—Mr. C. C. Baird, of the Dick Veterinary College, Edinburgh, has invented an india-rubber frog-pad for fitting into the heels of the shoes of horses, with the view of preventing the animals from slipping and falling on the causeway. The invention is exceedingly simple, and will be of great value to medical men and others who drive horses, especially on asphalt and in frosty weather. The pad can be removed in the evening and replaced in the morning. A set of pads, it may be added, is expected to wear out two sets of shoes. The trial of the invention, we believe, has fully borne out the statements of the inventor as to the merits of the apparatus.—*Medical Press.*

